# Understanding health care-seeking behaviour of the tribal population in India among those with presumptive TB symptoms

**DOI:** 10.1371/journal.pone.0250971

**Published:** 2021-05-20

**Authors:** Beena E. Thomas, Kannan Thiruvengadam, Raghavi S., Sudha Rani, Vetrivel S., Vikas Gangadhar Rao, Rajiv Yadav, Bhat J., Vijayachari Paluru, Anil Jacob Purthy, Tahziba Hussain, Anil Kumar Indira Krishna, Alex Joseph, Avi Kumar Bansal, Praveen Anand, Pradeep Das, K. R. John, Rekha Devi K., Sunish P., Rony Moral, Azhagendran S., Chandrasekaran V., Toteja G. S., Madhuchhanda Das, A. M. Khan, Harpreet Kaur

**Affiliations:** 1 Indian Council of Medical Research–National Institute for Research in Tuberculosis, Chennai, Tamil Nadu, India; 2 Indian Council of Medical Research–National Institute for Research in Tribal Health, Jabalpur, Madhya Pradesh, India; 3 Indian Council of Medical Research–Regional Medical Research Centre, Port Blair, Andaman and Nicobar Islands, India; 4 Pondicherry Institute of Medical Sciences, Puducherry, India; 5 Indian Council of Medical Research–Regional Medical Research Centre, Bhubaneshwar, Odisha, India; 6 School of Public Health, SRM Institute of Science and Technology, Kattankulathur, Tamil Nadu; 7 National JALMA Institute for Leprosy & Other Mycobacterial Diseases, Agra, India; 8 Desert Medicine Research Centre, Jodhpur, India; 9 Rajendra Memorial Research Institute of Medical Sciences, Patna, India; 10 Apollo Institute of Medical Sciences & Research, Chittoor, India; 11 Regional Medical Research Centre, Dibrugarh, India; 12 Rajendra Institute of Medical Sciences, Ranchi, Jharkhand, India; 13 Indian Council of Medical Research, New Delhi, India; ESIC Medical College & PGIMSR, INDIA

## Abstract

**Background and objectives:**

Understanding the drivers for care-seeking among those who present with symptoms of TB is crucial for early diagnosis of TB and prompt treatment, which will in turn halt further TB transmission. While TB is a challenge among the tribal population, little is known about the care-seeking behaviour and the factors influencing care-seeking behaviour among the tribal population across India.

**Methodology:**

This community-based descriptive study was carried out in 17 states of India across 6 zones, covering 88 villages from tribal districts with over 70% tribal population. The sample population included individuals ≥15 years old who were screened through an interview for symptoms suggestive of pulmonary TB (PTB), currently and/or previously on anti-TB treatment. Those with symptoms were then assessed on their health-seeking behavior using a semi-structured interview schedule.

**Results:**

Among 74532 eligible participants screened for symptoms suggestive of TB, 2675 (3.6%) were found to be presumptive TB cases. Of them, 659 (24.6%) sought care for their symptoms. While 48.2% sought care after a week, 19.3% sought care after one month or more, with no significant difference in the first point of care; 46.9% approaching a private and 46.7% a public facility. The significant factors influencing care-seeking behaviour were knowledge on TB (OR: 4.64 (3.70–5.83), p < 0.001), age<35 years (OR: 1.60 (1.28–2.00), p < 0.001), co-morbidities like asthma (OR: 1.80 (1.38–2.35), p < 0.001) and blood pressure (OR: 2.59 (1.75–3.85), p < 0.001), symptoms such as blood in sputum (OR: 1.69 (1.32–2.16), p < 0.001), shortness of breath (OR: 1.43 (1.19–1.72), p < 0.001) and weight loss (OR: 1.59 (1.33–1.89), p < 0.001). The cough was the most often reported symptom overall. There were gender differences in symptoms that prompted care-seeking: Males were more likely to seek care for weight loss (OR: 1.78 (1.42–2.23), p<0.001), blood in the sputum (OR: 1.69 (1.25–2.28), p<0.001), shortness of breath (OR: 1.49 (1.18–1.88), p<0.001) and fever (OR: 1.32 (1.05–1.65), p = 0.018). Females were more likely to seek care for blood in sputum (OR: 1.68 (1.10–2.58), p = 0.018) and shortness of breath (OR = 1.35, (1.01–1.82), p = 0.048). The cough did not feature as a significant symptom that prompted care-seeking.

**Conclusion:**

Delayed healthcare-seeking behaviour among those with symptoms presumptive of TB in the tribal population is a major concern. Findings point to differences across gender about symptoms that prompt care-seeking in this population. Gender-sensitive interventions with health system strengthening are urgently needed to facilitate early diagnosis and treatment among this population.

## Background

The global burden of TB has estimated 10 million people affected in 2019 and India shares 26% of it [1]. The major goal of the TB program in India is to reach TB elimination through the National TB Elimination Programme (NTEP) by 2025 [2]. Achieving these targets in the face of limited resources will require a focused inclusive approach, considering the diversity of the Indian population. This diversity includes a large tribal population that makes 8.6% of the Indian population which translates to 111 million people [3]. The tribal population contributes 9.8% to the total TB incidence in India [4], This group is categorized as one of the key affected populations in the National Strategic plan as they are scattered across India with limited access to health care, accentuated by low levels of awareness and they are highly influenced by distinctive social, cultural and economic factors [5–7].

If the goal of the NTEP needs to be achieved, the tribal population needs to be reached and the right focused interventions need to be adopted. One of the important areas in the context of TB apart from understanding the burden of TB among them in terms of prevalence is to gain insight into the health care-seeking behavior patterns and especially underlying barriers to care-seeking and treatment. Recent researches suggest that the delay between the onset of symptoms and first contact to the health care provider is one of the greatest contributors to ongoing TB mortality and incidence [8,9].

A meta-analysis by Thomas B, et al (2015) has estimated the prevalence of TB among the tribal population in India as 703 per 100,000 population, which is much more than the estimated national pooled prevalence of TB in the general population [10–12]. Although, TB is a major health problem among tribal communities; evidence of studies conducted on this population are rather limited and concentrated only in a few isolated groups mostly from central India [13–18]. The tribal population in India however is diverse and scattered across India with most of the tribal groups residing in remote areas.

While there are studies on the health-seeking behavior among TB patients and also chest symptomatic [19,20] in the general population but there is a dearth of information on the health-seeking behavior patterns and factors influencing care seeking among the tribal population who present symptoms that warrant care [19]. Thus understanding this becomes even more relevant given the remote geographical locations that this population resides in and the reality of limited access to healthcare.

The TB control program (RNTCP) has a tribal plan to cater to the tribal population. There are challenges in its implementation which include lack of understanding of their health care-seeking behaviour patterns. It is against this background that we present the findings of our nationwide study among the tribal population focusing on the health care-seeking behavior of those who have symptoms presumptive of TB. The findings of this study would help towards designing interventions which are more specific and need related for Tribal concentrated areas for better TB control.

## Methodology

This community-based study was part of a large nationwide study to estimate the prevalence of TB among the tribal population and to understand their health care-seeking behavior. The study was conducted between April 2015 and March 2020 among individuals (tribes) aged ≥15 years in tribal villages (clusters) which were selected using population proportional to the estimated size (PPES) method. A sample size of 63480 was estimated by assuming a disease prevalence of 387/100,000 population [21] precision of 15%, design effect of 1.3 [22] at 5% level of significance and a non-response of 10% [21].

A multistage cluster sampling design without replacement was adopted. The entire country was divided into 6 zones, each with two or more states: East, West, North, South, Central and North East. In each zone, tribal districts (with >70% tribal population) were listed along with the list of villages in these tribal districts. Once this list was complete, the villages (clusters) from these districts were selected based on PPES. A total of 88 villages were selected from 17 states of India. To achieve the sample size, a minimum of 800 individuals were selected from each selected village. In each village, streets were randomly selected and covered till the required sample size was achieved. If the required sample could not be achieved in a selected villages/clusters because they were smaller than the nearest listed village was selected to complete the sample size.

### Study participants

The total eligible tribal population covered in all these 88 villages was 92038, of which 74532 (81%) were screened for this study after obtaining informed consent. Reasons for non-inclusion were their unwillingness to provide consent and their non-availability at home even after three attempts were made to contact them.

The field investigators for the study were carefully selected from the concerned district and care was taken to select those who could speak the local language. The investigators were those with the graduates/post graduate degree in any science/social science or social work background. They underwent intense training which included how to approach tribal populations, screen for symptoms and conduct interviews.

Prior to the survey, planning visits to the districts and each of the villages were conducted by principal investigators and the team to meet the district officials and TB programme personnel for their approvals for the study. This was followed by visits to the tribal villages to meet with the influential people in the village and brief them on the purpose of the study and what it entails and the need for their cooperation and support in the conduct of the study. This included the heads of the village, panchayat leaders, and various representatives from the village that included tribal youth, men, and women. This was done through both individual meetings and group meetings.

The 74532 individuals were interviewed in their homes by the trained investigators. The interviews included two parts- the first part focused on socio-demographic details, alcohol and drug use, smoking history and self-reports on co-morbidities such as asthma, BP, diabetes, and malaria. and knowledge on TB. Details on smoking, alcohol consumption and drug use were assessed as an everyday practice (daily) with a positive or negative affirmation (Yes/No). Knowledge of TB was assessed with a 23 item set of questions that were scored and a median score of ≥6 were considered high and <6 considered low.

The second part of the schedule was only for those who reported TB symptoms referred to as a person with presumptive TB. He/she is one who presented with any of the following symptoms; persistent cough more than two weeks, cough with expectoration, fever, loss of appetite, blood in sputum, night sweats, weight loss, shortness of breath and excessive fatigue anytime within the last three months. Questions in this part were related to the action-taking behavior of those persons who reported TB symptoms. These details included details of when they sought care, choice of provider, the satisfaction of provider and the reasons for not seeking care.

### Data analysis

Responses were directly entered into the structured electronic form with the validation and logical constraints, developed using Open Data Kit (ODK) (opendatakit.org), an open-access software tool. During the field operation, collected data was reviewed for quality assurance and uploaded directly to the main server.

The data were described using frequency and percentages. The crude odds ratio was calculated and reported with 95% confidence interval, to identify the associations of care-seeking with the demographic variables, access to care, health system-related variables, alcohol and drug use, knowledge on TB, co-morbidities and symptoms using logistic regression. Chi-square test was used to test the difference in the proportion between the gender. A Multiple logistic regression analysis was performed to predict health-seeking behavior based on symptoms by gender. Significance was determined at p-value < 0.05. Statistical analysis was carried out using STATA version 16.1 (StataCorp, Texas, USA).

### Ethics statement

The study protocol was approved by the Scientific Advisory Committee and Institutional Ethics Committee of the Indian Council of Medical Research–National Institute for Research in Tuberculosis, Chennai (NIRT-IEC-ID:2014005) Written informed consent was obtained from all study participants. For participants between 15–18 years, assent was obtained from the individuals and written informed consent was obtained from parents/guardians/caretakers/next to kin. Children below 15 years were not included in the study. The privacy and confidentiality of all participants were ensured.

## Results

### Prevalence of persons with presumptive TB

**Fig 1:**
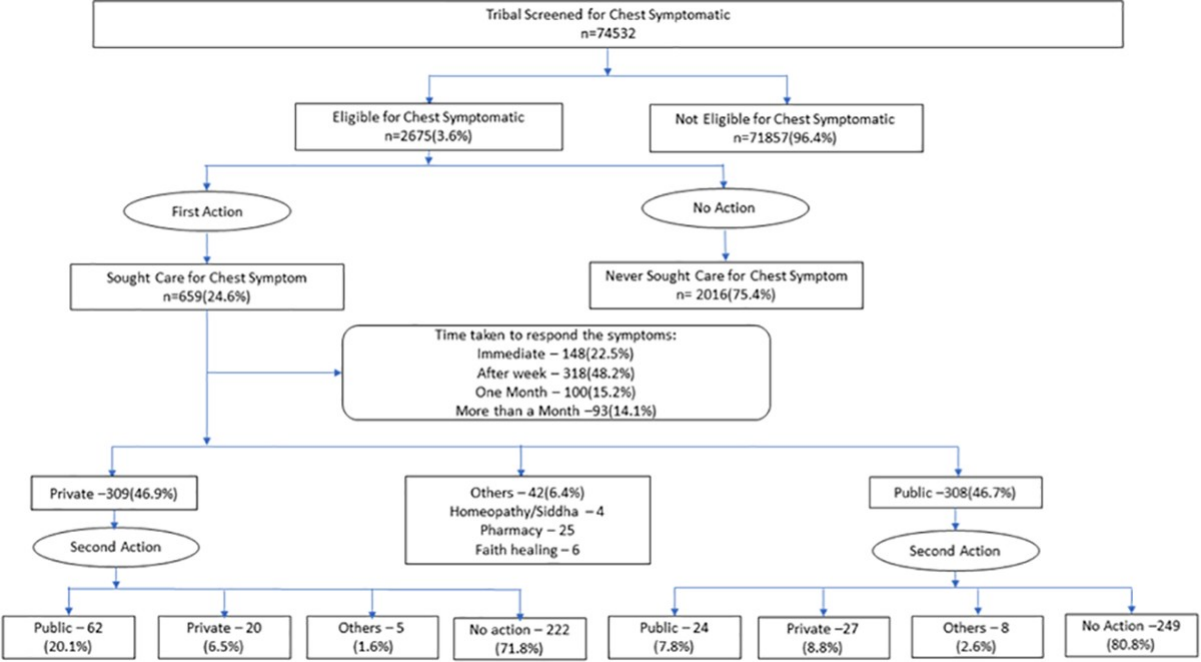
Care seeking behaviour of chest symptomatic.

The total eligible tribal population in these 88 clusters was 92038, of which 74532 were enrolled for the study. The social and demographic profile of the population of 74532 has been presented in Table 1. Of the 74532 study population, 2675 (3.6%) reported TB symptoms and were considered as persons with presumptive TB as defined by the Revised National Tuberculosis Control Program (RNTCP) [23]. We present the health care seeking behavior of this population (n = 2675) (Fig 1).

**Table 1:** Demographic details of the study population and the persons with presumptive TB.

	Population (N = 74532)	%	Presumptive TB Patients(n = 2675)	%
**Age**				
15–34	38390	51.5%	730	27.3%
35–54	22973	30.8%	974	36.4%
≥55	13169	17.7%	971	36.3%
**Sex**				
Female	39000	52.3%	1031	38.5%
Male	35532	47.7%	1644	61.5%
**Occupation**				
Others	9888	13.3%	93	3.5%
Daily Labourer	64644	86.7%	2582	96.5%
**Education**				
Literate (any formal education)	42884	57.5%	1132	42.3%
Illiterate (neither can read or write)	31648	42.5%	1543	57.7%
**BMI**				
≥18.5	53961	72.4%	1487	55.6%
<18.5	20571	27.6%	1188	44.4%
**Alcohol Consumption**				
No	59499	79.8%	1809	67.6%
Yes	15033	20.2%	866	32.4%
**Smoking**				
No	66803	89.6%	2012	75.2%
Yes	7729	10.4%	663	24.8%
**Substance use**				
No	69584	93.4%	2391	89.4%
Yes	4948	6.6%	284	10.6%
**Knowledge on TB**				
Have not heard about TB	36130	48.5%	1107	41.4%
Low (<6)	17063	22.9%	680	25.4%
High (≥6)	21339	28.6%	888	33.2%
**Long Distances from Health facility**				
No	49296	66.1%	1697	63.4%
Yes	25236	33.9%	978	36.6%

### Action taking behaviour of persons with presumptive TB and facility they sought care from

At the time of the interview, 659 (24.6%) of persons with presumptive TB had sought care for their symptoms and 2016 (75.4%) did not seek care. Overall, 308 (46.7%) approached a government healthcare facility as their first point of care while 309 (46.9%) approached a private facility. Remaining individuals i.e. 4 (9.5%), 25 (59.5%) and 6 (14.3%) approached AYUSH doctors, pharmacy and traditional healers, respectively (Fig 1).

### The interval between onset of symptoms and care

Out of the 659 who sought care, 148 (22.5%) took action within a week of presenting symptoms, 318 (48.2%) after a week and 193 (19.3%) took action after a month or more (Fig 1). There was no difference in the action taking behavior across gender.

### Reasons for not seeking care

**Fig 2:**
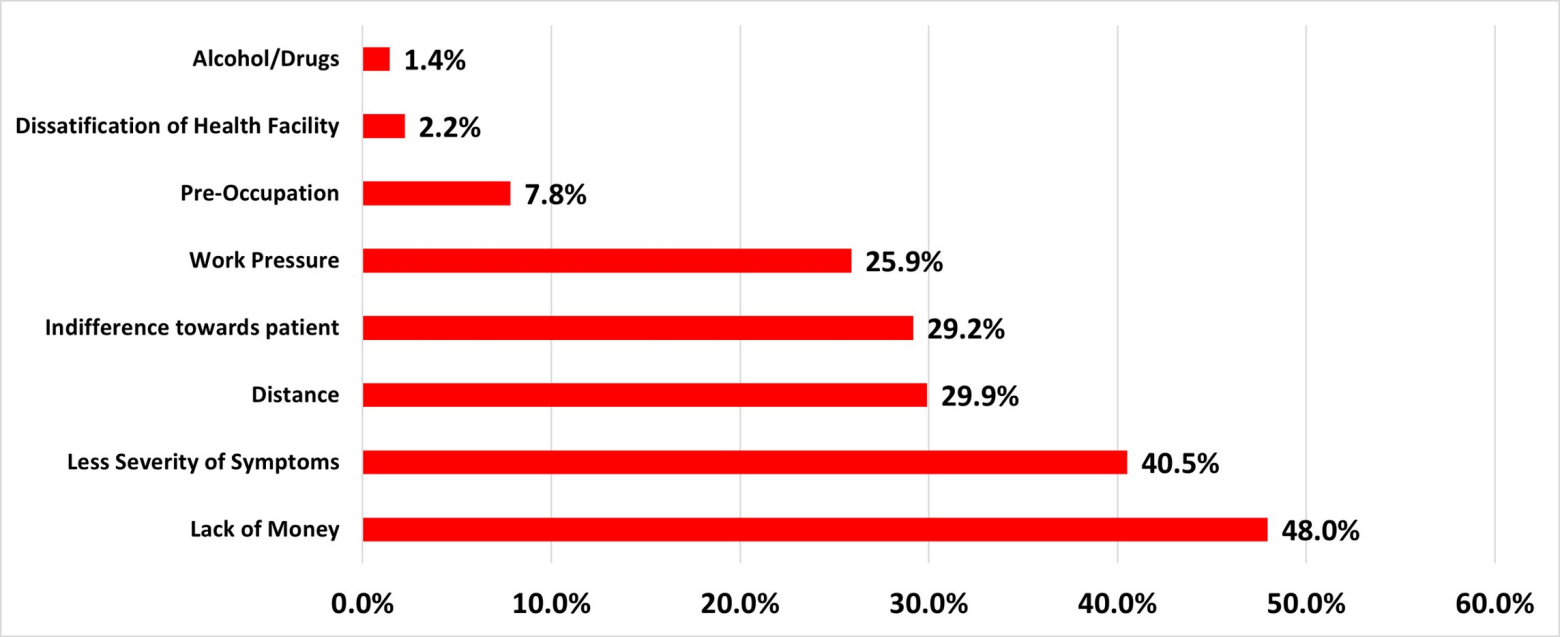
Reasons for not seeking care.

The main reasons for not seeking care were lack of money (48.0%), symptoms not severe (40.5%), long distances to health facility (29.9%) and indifferent behaviour of the healthcare providers (29.2%) (Fig 2). There was no difference across gender except in the severity of symptoms with men reporting less severity of symptoms as a reason for not seeking care (p = 0.02).

### Factors influencing care-seeking behaviour

The factors influencing care seeking were knowledge on TB (OR: 4.64 (3.70–5.83), p<0.001), age <35 (OR: 1.60, (1.28–2.00), p<0.001), literacy (OR: 1.38, (1.15–1.64), p<0.001), substance abuse (OR: 2.70, (1.86–3.92), p<0.001), access to health facility (OR: 1.54, (1.27–1.86), p<0.001), expenditure incurred (OR: 1.58, (1.30–1.91), p<0.001), history of blood pressure (OR: 2.59 (1.75–3.85), p<0.001), asthma (OR: 1.80 (1.38–2.35), p<0.001) and symptoms like blood in sputum (OR: 1.69 (1.32–2.16), p<0.001), fever (OR: 1.22 (1.02–1.46), p = 0.030), tiredness (OR: 1.27 (1.06–1.52), p = 0.008), HIV (OR: 3.56 (2.02–6.28), p<0.001), weight loss (OR: 1.59 (1.33–1.89), p<0.001) and shortness of breath (OR: 1.43 (1.19–1.72), p<0.001) (Table 2).

**Table 2:** Factors influencing Chest Symptomatic patients to seek care (n = 2675).

Factors	Seek Care		OR (95% CI)	p Value
	No (n = 2016)	Yes (n = 659)		
**Age**				
15–34	521 (71.4)	209 (28.6)	1.60 (1.28–2.00)	<0.001
35–54	719 (73.8)	255 (26.2)	1.41 (1.14–1.75)	0.001
≥55	776 (79.9)	195 (20.1)	1.00	
**Sex**				
Female	781 (75.8)	250 (24.2)	1.00	
Male	1235 (75.1)	409 (24.9)	1.03 (0.86–1.24)	0.713
**Occupation**				
Unemployed	60 (64.5)	33 (35.5)	1.72 (1.11–2.65)	0.015
Employed	1956 (75.8)	626 (24.2)	1.00	
**Education**				
Literate	814 (71.9)	318 (28.1)	1.38 (1.15–1.64)	<0.001
Illiterate	1202 (77.9)	341 (22.1)	1.00	
**BMI**				
≥18.5	1136 (76.4)	351 (23.6)	1.00	
<18.5	880 (74.1)	308 (25.9)	1.13 (0.95–1.35)	0.166
**Alcohol Consumption**				
No	1371 (75.8)	438 (24.2)	1.00	
Yes	645 (74.5)	221 (25.5)	1.07 (0.89–1.29)	0.463
**Smoking**				
No	1524 (75.7)	488 (24.3)	1.00	
Yes	492 (74.2)	171 (25.8)	1.09 (0.89–1.33)	0.426
**Substance use**				
No	1765 (73.8)	626 (26.2)	2.70 (1.86–3.92)	<0.001
Yes	251 (88.4)	33 (11.6)	1.00	
**Knowledge on TB**				
Have not heard about TB	976 (88.2)	131 (11.8)	1.00	
Low (<6)	493 (72.5)	187 (27.5)	2.83 (2.2–3.62)	<0.001
High (≥6)	547 (61.6)	341 (38.4)	4.64 (3.7–5.83)	<0.001
**Long Distance**				
No	1231 (72.5)	466 (27.5)	1.54 (1.27–1.86)	<0.001
Yes	785 (80.3)	193 (19.7)	1.00	
**Poor attitude of health care workers**				
No	1621 (75.2)	536 (24.8)	1.06 (0.85–1.33)	0.601
Yes	395 (76.3)	123 (23.7)	1.00	
**Lack of services**				
No	1795 (75)	598 (25)	1.21 (0.90–1.63)	0.216
Yes	221 (78.4)	61 (21.6)	1.00	
**Cost incurred**				
No	1251 (72.5)	475 (27.5)	1.58 (1.30–1.91)	<0.001
Yes	765 (80.6)	184 (19.4)	1.00	
**Cough**				
No	199 (74)	70 (26)	1.00	
Yes	1817 (75.5)	589 (24.5)	0.92 (0.69–1.23)	0.578
**Expectoration**				
No	727 (75.3)	239 (24.7)	1.00	
Yes	1289 (75.4)	420 (24.6)	0.99 (0.83–1.19)	0.924
**Chest Pain**				
No	548 (73.7)	196 (26.3)	1.00	
Yes	1468 (76)	463 (24)	0.88 (0.73–1.07)	0.203
**Fever**				
No	1246 (76.8)	376 (23.2)	1.00	
Yes	770 (73.1)	283 (26.9)	1.22 (1.02–1.46)	0.030
**Loss of Aptitude**				
No	1236 (76.7)	376 (23.3)	1.00	
Yes	780 (73.4)	283 (26.6)	1.19 (0.99–1.43)	0.053
**Blood in sputum**				
No	1794 (76.7)	545 (23.3)	1.00	
Yes	222 (66.1)	114 (33.9)	1.69 (1.32–2.16)	<0.001
**Night Sweat**				
No	1555 (75.2)	513 (24.8)	1.00	
Yes	461 (75.9)	146 (24.1)	0.96 (0.78–1.19)	0.705
**Weight loss**				
No	1255 (78.9)	336 (21.1)	1.00	
Yes	761 (70.2)	323 (29.8)	1.59 (1.33–1.89)	<0.001
**Shortness of breath**				
No	1408 (77.6)	407 (22.4)	1.00	
Yes	608 (70.7)	252 (29.3)	1.43 (1.19–1.72)	<0.001
**Tiredness**				
No	964 (77.7)	276 (22.3)	1.00	
Yes	1052 (73.3)	383 (26.7)	1.27 (1.06–1.52)	0.008
**Diabetes**				
No	1992 (75.5)	648 (24.5)	1.00	
Yes	24 (68.6)	11 (31.4)	1.41 (0.69–2.89)	0.350
**BP**				
No	1958 (76.2)	612 (23.8)	1.00	
Yes	58 (55.2)	47 (44.8)	2.59 (1.75–3.85)	<0.001
**HIV**				
No	1993 (75.9)	633 (24.1)	1.00	
Yes	23 (46.9)	26 (53.1)	3.56 (2.02–6.28)	<0.001
**Asthma**				
No	1840 (76.6)	562 (23.4)	1.00	
Yes	176 (64.5)	97 (35.5)	1.80 (1.38–2.35)	<0.001
**Malaria**				
No	1793 (75)	599 (25)	1.24 (0.92–1.68)	0.157
Yes	223 (78.8)	60 (21.2)	1.00	

Values were presented as count and percentages; Odds ratio (OR) was calculated using logistic regression.

### Symptoms reported overall and gender wise (N = 2675)

Overall, the symptoms reported were cough (89.9%), chest pain (72.2%), cough with expectoration (63.9%), tiredness (53.6%), weight loss (40.5%), loss of appetite (39.7%), fever (39.4%), shortness of breath (32.1%), night sweat (22.7%), and blood in sputum (12.6%).

Gender wise the symptoms reported among women and men were cough (90.7% vs 88.8%), chest pain (72.6% vs 72.0%), cough with expectoration (60.6% vs 65.9%), tiredness (53.4% vs 53.8%), fever (43.3% vs 36.9%), weight loss (39.1% vs 41.4%), shortness of breath (32.1% vs 32.2%), loss of appetite (41.5% vs 38.6%), night sweat (22.0% vs 23.1%) and blood in sputum (10.4% vs 13.9%).

### Gender difference in symptoms influencing health care seeking behaviour (N = 2675)

Symptoms that significantly influenced health care seeking behaviour among males were blood in sputum (aOR: 1.51 (1.10–2.09), p = 0.012), weight loss (aOR: 1.80 (1.36–2.39), p<0.001) and shortness of breath (aOR: 1.37 (1.04–1.80), p = 0.026). Among the females, blood in sputum (aOR: 1.68 (1.08–2.63), p = 0.022) was the significant symptom which influenced care seeking. Symptoms like cough, chest pain, expectoration, loss of appetite and night sweats did not significantly prompt care seeking (Table 3).

**Table 3:** Symptoms influencing persons with presumptive TB to seek care by gender.

Symptoms	Female					Male				
	Action	OR	p-Value	aOR	p-Value	Action	OR	p-Value	aOR	p-Value
	Taken	(95% CI)		(95% CI)		Taken	(95% CI)		(95% CI)	
**Cough**										
No	27 (23.3)	1.00		1.00		43 (28.1)	1.00		1.00	
Yes	223 (24.4)	1.06	0.795	1.11	0.678	366 (24.5)	0.83	0.333	0.76	0.169
		(0.67–1.68)		(0.68–1.8)			(0.57–1.21)		(0.51–1.13)	
**Expectoration**										
No	103 (25.4)	1.00		1.00		136 (24.3)	1.00		1.00	
Yes	147 (23.5)	0.9	0.498	0.76	0.110	273 (25.2)	1.05	0.690	1.06	0.645
		(0.68–1.21)		(0.55–1.06)			(0.83–1.33)		(0.82–1.39)	
**Chest Pain**										
No	77 (27.2)	1.00		1.00		119 (25.8)	1.00		1.00	
Yes	173 (23.1)	0.8	0.173	0.69	0.032	290 (24.5)	0.93	0.584	0.79	0.086
		(0.59–1.1)		(0.49–0.97)			(0.73–1.19)		(0.61–1.03)	
**Fever**										
No	138 (23.6)	1.00		1.00		238 (23.0)	1.00		1.00	
Yes	112 (25.1)	1.09	0.572	1.09	0.571	171 (28.2)	1.32	0.018	1.28	0.065
		(0.82–1.45)		(0.8–1.5)			(1.05–1.65)		(0.99–1.66)	
**Loss of Aptitude**										
No	140 (23.2)	1.00		1.00		236 (23.4)	1.00		1.00	
Yes	110 (25.7)	1.14	0.359	1.04	0.841	173 (27.2)	1.23	0.079	1.15	0.348
		(0.86–1.53)		(0.73–1.47)			(0.98–1.54)		(0.86–1.53)	
**Blood in sputum**										
No	214 (23.2)	1.00		1.00		331 (23.4)	1.00		1.00	
Yes	36 (33.6)	1.68	0.018	1.68	0.022	78 (34.1)	1.69	<0.001	1.51	0.012
		(1.1–2.58)		(1.08–2.63)			(1.25–2.28)		(1.1–2.09)	
**Night Sweat**										
No	197 (24.5)	1.00		1.00		316 (25.0)	1.00		1.00	
Yes	53 (23.3)	0.94	0.720	0.79	0.242	93 (24.5)	0.97	0.835	0.68	0.017
		(0.66–1.33)		(0.54–1.17)			(0.74–1.27)		(0.5–0.93)	
**Weight loss**										
No	140 (22.3)	1.00		1.00		196 (20.4)	1.00		1.00	
Yes	110 (27.3)	1.31	0.068	1.29	0.155	213 (31.3)	1.78	<0.001	1.8	<0.001
		(0.98–1.75)		(0.91–1.82)			(1.42–2.23)		(1.36–2.39)	
**Shortness of breath**										
No	157 (22.4)	1.00		1.00		250 (22.4)	1.00		1.00	
Yes	93 (28.1)	1.35	0.048	1.33	0.107	159 (30.1)	1.49	<0.001	1.37	0.026
		(1.01–1.82)		(0.94–1.87)			(1.18–1.88)		(1.04–1.8)	
**Tiredness**										
No	106 (22.1)	1.00		1.00		170 (22.4)	1.00		1.00	
Yes	144 (26.1)	1.25	0.130	1.15	0.452	239 (27.0)	1.29	0.029	1.01	0.940
		(0.94–1.66)		(0.8–1.66)			(1.03–1.61)		(0.75–1.37)	

Values were presented as count and percentages.

Odds ratio (OR) and adjusted Odds ratio (aOR) were calculated using logistic regression.

## Discussion

A prevalence of presumptive TB among the tribal population (4%) is similar to the prevalence of presumptive TB amongst the general population [20]. The strength of our study is the salient findings with regard to the care-seeking behavior among these individuals. Firstly, contrary to George O et al (2013) findings, 75% of the participants in the current study did not seek care for their symptoms [24].

Secondly, our study helps gain insight into the reasons for not seeking care among the tribal population, which were lack of money, symptoms not being severe enough, distance to health facilities and dissatisfaction with the healthcare providers. These findings are in concordance with other studies that reported lack of money, non-severity of symptoms and long-distance to health facility [5,20,24–27]. The reasons were similar across gender except that more males reported less severity of symptoms as a reason for not seeking care. Apart from the manifold challenges faced by the tribal population from being located in remote hard to reach areas, impediments such as access to care and poor care from the health care providers is worrisome.

Thirdly, our study reports that only a small percentage sought immediate care for their TB symptoms with more than one-third of them seeking care after a month or more. These findings seem to echo the findings of a study in a tribal population in Rayagada, Odisha, that reported only 5% of the patients sought care within 2 weeks and more than 60% of participants delayed seeking care for more than a month after onset of symptoms [28]. A mean delay to diagnosis has been reported as 36.5 days in another study from Odisha among the tribal population [26]. This delay in seeking care impedes early diagnosis, thereby increasing the risk of transmitting the disease among this population. Recent research suggests that the delay between the onset of symptoms and first contact with a provider to seek care is one of the greatest contributors to ongoing TB mortality and incidence [8,9]. Reports of modeling indicate that, in India, if the average care-seeking delay for those infected with TB was reduced by 25%, TB mortality would be reduced by roughly 6% and the incidence of new cases would similarly decline [8].

Fourthly, it is also interesting that in our study almost equal numbers sought care in public and private health care facilities and seeking care among traditional healers, who are largely believed to be the first point of care among the tribal population was negligible. This is different from the general population which reports that private care facilities are accessed more as compared to public facilities [20,24,27,29]. The reason for this difference could be the fact that private healthcare facilities are present in less number in tribal areas and they are not the preferred choice. This points out the need to strengthen government health facilities in tribal areas to further minimize the reliance of these communities on private healthcare that is often unaffordable which could result in discontinuation of care.

Apart from these findings, our study highlights the factors influencing care-seeking. Knowledge on TB was observed as the most significant factor influencing care-seeking behaviour among persons with TB symptoms. This finding is supported by other studies in the Indian context [18,30,31]. Nearly 50% of the population with and without symptoms had not even heard of TB with another quarter of the respondents having low awareness. Poor awareness of TB among the tribal population heightens the need for tribal friendly TB sensitization programs. Although IEC materials on TB available but its modification in terms of the local language and cultural acceptability is required to address this gap [29] and cater to the culture of the tribal population and made available in their dialects/languages.

Other significant factors influencing care-seeking were alcohol and substance abuse with one-third of the presumptive TB patients reporting daily alcohol use and 17% reporting substance use. Alcohol is most often home-brewed and consumed by both men and women. The common drugs used are marijuana which is widely grown in some areas and another was ‘apparat’ (betel nut, cardamom, lime, and catechu), which is available as sachets and chewed like tobacco. While alcohol use and substance use is an accepted practice among the tribal population, the fact that this could impede seeking healthcare especially for those with presumptive TB symptoms needs consideration. Perhaps the consumption of alcohol or drugs is perceived as a remedy for symptoms which needs to be explored further. The problems associated with alcohol and drug use among the tribal population have also been reported in earlier studies [32–34]. These findings of the current study stresses that there is a need to develop innovative alcohol and drug use intervention strategies that promote early care-seeking. While alcohol and drug use were barriers to seeking care, interestingly those with low BMI were more likely to seek care. This is further reiterated in the finding which emerged from this study that loss of weight was one of the dominant symptoms that promoted care-seeking.

It was also interesting to note that self-reports of co-morbidities like asthma and BP were influential factors for seeking care. This points out the fact that the sensitization about non-communicable diseases (NCDs) has led to the tribal population seeking care for NCDs. While communicable diseases like malaria and TB continue to be rampant among tribal communities in India, changing lifestyles with many of the tribal population having increased mobility to cities have also led to a rise in the prevalence of NCDs like cancer, sickle cell anemia, hypertension and diabetes [30,31,35–38]. Perhaps this calls attention to the need for more TB sensitization and screening for those who are affected with NCDs for early diagnosis of TB.

Concerning gender, our study findings reports that more men among the tribal population are taking action for their TB symptoms as compared to women. The first action that women resort to is home remedies and only after a prolonged duration of cough they seek care [39]. This is a behavior pattern among women in general considering the burden they shoulder as caregivers which impede them from leaving home to seek care in a health facility till the symptoms become unbearable.

Finally, our study points to the gender differences observed in seeking care concerning the dominant symptoms that influenced care-seeking. Overall, shortness of breath was the most dominant symptom prompting care followed by blood in sputum, weight loss and cough with expectoration. However, it is interesting to note the significant differences in this expression among females and males. Among females shortness of breath was the most dominant symptom that prompted to seek care and among males it was blood in sputum. While a cough is a symptom recognized by the TB control program as an important symptom that classifies a person as being a presumptive TB patient, our results point to the fact that despite a large number of the respondents suffering from cough (93%), they did not seek care for their symptoms. Perhaps this is because the cough is usually associated with either smoking or a mild symptom and only with severity becomes a symptom that prompts to seek care. The TB control programme across the globe emphasizes cough being a very important symptom of TB. A study from China reports a longer delay for cough as compared to cough with hemoptysis [25]. A study from Ethiopia reports that those with cough less than 30 days were less likely to seek care. Furthermore, among the tribal population symptoms that do hinder them from not doing their routine work are not considered severe [7,40]. This points to the need for gender-focused TB IEC strategies as well as timely intervention for those with presumptive TB who present with what they consider important symptoms which may not be the same as highlighted by the TB control programme.

## Conclusion and recommendations

Health care-seeking behaviour among persons with presumptive TB in the tribal population is worrisome and needs to be strengthened. TB sensitization and advocacy efforts require a better understanding of the symptoms that promote timely care-seeking for early diagnosis and treatment initiation among this population. This is often overlooked as the perception of the symptoms that warrant care among the tribal population may differ from the general population. These interventions need to be tribal friendly and gender-sensitive through community engagement utilizing tribal community representatives that include youth, traditional leaders, influential men and women and elected leaders. This would help for better acceptability and ownership within the community for TB control among them. This strategy could focus not only on promoting TB awareness that caters to the tribal community but also on the referral of individuals with presumptive TB through the community representatives to health centers. This entails linkage to the health system which needs to be built into this intervention as without this networking this exercise may not prove productive.

## Limitations

The limitations of this study is that this was a cross-sectional study which limits the generalizability of the findings of the study as the regression analysis becomes week and it needs to be explored further. The symptoms of TB are self-reported and they were not confirmed by any cross-checking methods or laboratory tests. The clusters were chosen from districts with over 70% tribal population, data from many PVTG (Particularly Vulnerable Tribal Group) who live in extremely remote areas were not part of the selected >70% tribal districts. Considering that this is a vulnerable group, more research to generate information on healthcare-seeking behaviour and challenges in healthcare from this group would be beneficial for the TB control programme.

The uniqueness of this study is that it is the first study to assess the health care seeking behavior of TB among tribal population at the national level across different geographic regions. The sampling was rigorous and scientifically done to ensure that the population was representative from tribal districts clearly defined as those districts with over 70 percent tribal population.

## Supporting information

S1 Appendix Dataset for understanding health care-seeking behaviour of the tribal population in India among those with presumptive TB symptoms. (RAR)

S2 Appendix Data dictionary accompanying the dataset for understanding health care-seeking behaviour of the tribal population in India among those with presumptive TB symptoms. (TXT)
